# Tyrosinase inhibition and antioxidant properties of *Asphodelus microcarpus* extracts

**DOI:** 10.1186/s12906-016-1442-0

**Published:** 2016-11-09

**Authors:** Amalia Di Petrillo, Ana Maria González-Paramás, Benedetta Era, Rosaria Medda, Francesca Pintus, Celestino Santos-Buelga, Antonella Fais

**Affiliations:** 1 Department of Life and Environmental Sciences, University of Cagliari, SS 554, Bivio per Sestu, I-09042 Monserrato, Cagliari, Italy; 2 Grupo de Investigación en Polifenoles, Unidad de Nutrición y Bromatología, Facultad de Farmacia, Universidad de Salamanca, Salamanca, Spain

**Keywords:** Antioxidants, *Asphodelus microcarpus*, B16F10 melanoma cells, Flavonoids, Polyphenols, Tyrosinase inhibitors

## Abstract

**Background:**

*Asphodelus microcarpus* belongs to the family Liliaceae that include several medicinal plants. In the traditional medicine plants of the genus *Asphodelus* are used to treat skin disorders such as ectodermal parasites, psoriasis, microbial infection and for lightening freckles. In order to find novel skin depigmenting agents, the present work was carry out to evaluate antioxidant activity and tyrosinase inhibitory potential of leaves, flowers and tubers extracts of *A. microcarpus*. The phytochemical composition of the active extract was also evaluated.

**Methods:**

Three different extracts (water, methanol and ethanol) from leaves, flowers and tubers of *A. microcarpus* were evaluated for their inhibitory effect on tyrosinase activity using l-3,4-dihydroxyphenylalanine (l-DOPA) as substrate. Inhibition of cellular tyrosinase activity and melanin production was also investigated in melanoma B16F10 cells. Antioxidant activity, total phenolic and flavonoids contents were determined using standard in vitro methods. HPLC-DAD-MS was used to identify phenolic profile of the active extract.

**Results:**

The results showed that all extracts have a direct inhibitory anti-tyrosinase activity, with ethanolic extract from flowers (FEE) exhibiting the stronger effect. Kinetic analysis revealed that FEE acts as an uncompetitive inhibitor with a Ki value of 0.19 mg/mL. The same effect was observed in murine melanoma B16F10 cells. Cellular tyrosinase activity as well as melanin content were reduced in FEE-treated cells. The results were comparable to that of the standard tyrosinase inhibitor (kojic acid). Furthermore, the same extract showed the highest antioxidant activity and an elevated levels of total phenolics and flavonoid content. Eleven phenolic components were identified as chlorogenic acid, luteolin derivates, naringenin and apigenin.

**Conclusions:**

Our findings showed that FEE from *A. microcarpus* inhibits tyrosinase and exerted antimelanogenesis effect in B16F10 cells. This extract also showed the highest scavenging activity, which could be mainly attributed to its high levels of total polyphenols and flavonoids. These results suggest that *A. microcarpus* has a great potential as sources of bioactive compounds which could be used as depigmenting agents in skin disorders.

## Background

Melanin is a pigment which plays an important role in the protection against UV damage and represents an important defense system of the skin against harmful factors. Despite its advantages, melanin is also involved in abnormal pigmentation and melanoma; therefore, different approaches to the study of skin disorders has been developed [1–3].

Tyrosinase (EC 1.14.18.1) is the key enzyme in the first two steps of melanin biosynthesis, catalyzing the hydroxylation L-tyrosine to the 3,4-dihydroxyphenylalanine (DOPA) and the oxidation of DOPA to dopaquinone.

Overproduction and accumulation of melanin occur in several skin disorders including solar melanosis, ephelides, melasma, senile lentigos and postinflammatory hyperpigmentation [4]. Since tyrosinase is the limiting step enzyme in melanogenesis, its inhibitors have become increasingly important as depigmenting agents in hyperpigmentation disorders.

However, currently available tyrosinase inhibitors suffer from toxicity and/or a lack of efficacy and there is a constant quest for better inhibitors from natural sources as they are expected to be free of harmful side effects [5, 6].

Several investigations have been done for identification of many naturally occurring substances in higher plants with antioxidant and other protective biochemical functions. The raw extracts or chemical constituents might be used for treatment of various human diseases [7]. Reactive oxygen species are considered as a major contributor to age-related symptoms and pathogenesis of many diseases. The skin is also vulnerable to oxidative stress, and exposure to repeated oxidative stress contributes to its aging [8]. Many synthetic antioxidants have potential hazards to health [9], therefore, there is a tendency to develop and utilize effective natural antioxidants to reduce the health risks.

In the traditional medicine, plants of genus *Asphodelus* are used to treat skin disorders, as well as ectodermal parasites, psoriasis, microbial infection and for lightening freckles [10, 11]. Various biological activities have been reported for *A. microcarpus*, [12, 13]. Phytochemical studies on *A. microcarpus* revealed the presence of lipids, carbohydrates, sterols, anthraquinones and arylcoumarins [13, 14]. It is well known that the last two compounds have tyrosinase inhibitory activity [15–17] and plant extracts with antimelanogenic activity typically possess polyphenols such as flavonoids, which are usually the factors responsible for the activities in plant extracts [18]. The aim of this study was to investigate the inhibitory activity of three different extracts of *A. microcarpus* on tyrosinase activity and on melanogenesis in B16F10 melanoma cells. In addition, total phenols, flavonoids contents and antioxidant capacities of the extracts have also been analyzed.

## Methods

### Reagents

All chemicals for antioxidant and enzyme activity were obtained as pure commercial products from Sigma Chemical Co (St. Louis, MO, USA) and used without further purification. HPLC-grade acetonitrile was obtained from Merck KgaA (Darmstadt, Germany) and formic acid was purchased from Prolabo (VWR International, France). Water was treated in a Milli-Q water purification system (TGI Pure Water Systems, USA). The phenolic compounds standards (5-O-caffeoylquinic acid, luteolin-6-C-glucoside, luteolin-7-O-glucoside and apigenin) were from Extrasynthese (Genay, France).

### Plant material

*Asphodelus microcarpus* subsp. *microcarpus* Salzm. et Viv. leaves, flowers and tubers (L, F and T respectively) were collected in southern Sardinia (Quartu Sant’Elena, Cagliari, Italy). The GPS coordinates were 39° 22′41.5″ N and 09° 19′62.3″ E. The plant was identified by Dr. Cecilia Loi, Professor of Plant Taxonomy, University of Cagliari, Italy. A voucher specimen (1405/16 Herbarium CAG) has been deposited in the Life and Environmental Sciences Department.

Plant materials were washed with deionized water, frozen at −80 °C and then lyophilized in intact condition. The dried plant was stored at −80 °C until required.

### Extraction procedure

The lyophilized plant materials (1 g) were extracted in 10 mL of water (AE, aqueous extract) or ethanol (EE, ethanol extract) or methanol (ME, methanol extract) for 24 h at room temperature under continuous stirring. After filtration, ethanol or methanol extracts were diluted 10-fold with water and then all extracts were lyophilized. Dried powders (1 mg) were dissolved in 1 mL of the apposite solvent (water or 1 % ethanol:water or 1 % methanol:water for AE, EE and ME respectively) before use. For HPLC–DAD–ESI/MS analyses dried extract was dissolved in 1 mL of 0.1 % formic acid:acetonitrile (70:30, v/v) and filtered through a 0.22 μm disposable LC filter disk for HPLC analysis.

### Antioxidant assays

In each extract total free radical-scavenging molecules were determined by ABTS^+^ (2,2′-azinobis-(3-ethylbenzothiazoline-6-sulfonic acid) and DPPH (2,2-diphenyl-1-picrylhydrazyl) methods as previously reported [19, 20]. For both free radical methods, 6-hydroxy-2,5,7,8-tetramethylchromane-2-carboxylic acid (Trolox) was used as standard reference. The concentration range of extract used for the antioxidant tests was 0–0.3 mg/mL.

The activity was expressed as concentration of sample necessary to give a 50 % reduction in the original absorbance (IC_50_).

### Determination of the total polyphenols and flavonoids

Total content of polyphenols and flavonoids in the extracts was determined as previously reported [21]. Polyphenol concentration was calculated using gallic acid as a referred standard and was expressed as mg of gallic acid equivalent (GAE) per 1 g of dry weight (dw). Flavonoid concentration was expressed as mg of quercetin equivalent (QE) per 1 g of dry extract.

### Mushroom tyrosinase inhibition assay

Tyrosinase inhibition assays were performed with l-DOPA as substrate. The reaction mixture (1000 μL) contained 685 μL of phosphate buffer (0.05 M, pH 6.5), 15 μL of mushroom tyrosinase (2500 U mL^−1^), 200 μL of plant extract solution and 100 μL of 5 mM l-DOPA. After the addition of l-DOPA the reaction was immediately monitored at 492 nm for dopachrome formation in the reaction mixture. Kojic acid was used as a positive control. The concentration range of extract used for the mushroom tyrosinase inhibition assay was 0–0.3 mg/mL. Each measurement was made in triplicate. The IC_50_ value, a concentration giving 50 % inhibition of tyrosinase activity, was determined by interpolation of concentration-response curves.

### Cell viability

B16F10 mouse melanoma cells (CRL-6475) were purchased from the American Type Culture Collection (ATCC, Manassas, VA, USA). The cells were cultured in Dulbecco’s Modified Eagle Medium (DMEM) supplemented with 10 % fetal bovine serum (FBS, Gibco, NY, USA), and 1 % penicillin/streptomycin at 37 °C in a humidified atmosphere with 5 % CO_2_. The colorimetric 3-(4,5-dimethylthiazol-2-yl)-2,5-diphenyltetrazolium bromide (MTT) assay was performed to determine cell viability [22].

### α-MSH treatment

B16F10 cells were seeded in 6-well plates (10^5^ cells/mL). After 24 h, the medium was substituted by fresh one supplemented with 100 nM α-melanocyte stimulating hormone (*α*-MSH) and different concentration of plant extract (0.05-0.15 mg/mL) and incubated for 48 h. Cells treated with 100 nM *α*-MSH and kojic acid were used as positive control and for comparing the inhibitory strength of the extracts.

### Intracellular tyrosinase activity and melanin content assay

The tyrosinase activity and melanin content in B16F10 cells were performed following a previously described method [23]. *α*-MSH-stimulated cells were plated in 60*π*-dishes (10^5^ cells/mL) and incubated for 48 h in absence or presence of sample (0.05–0.15 mg/mL). The protein content of cellular lysates were calculated by the Bradford method using BSA as a standard [24].

### l-DOPA staining assay

The DOPA-staining assay was performed as previously reported [23]. B16F10 cells were treated for 48 h with either *α*-MSH alone or *α*-MSH plus flowers extracts at different concentration or kojic acid (100 or 150 μg/mL) as positive control. After treatment, cells were lysed and protein extracts (5 μg) were analysed by 8 % SDS-polyacrylamide gel electrophoresis. After staining with DOPA, tyrosinase activity was visualized in the gel as dark melanin-containing bands.

### HPLC-DAD-ESI/MS analyses

The ethanolic extract of *A. microcarpus* flowers was analyzed using a Hewlett-Packard 1200 chromatograph (Agilent Technologies, Waldbronn, Germany) equipped with a binary pump and a diode array detector (DAD) coupled to an HP Chem Station (rev. A.05.04) data-processing station. The HPLC system was connected via the DAD cell outlet to an API 3200 Qtrap (Applied Biosystems, Darmstadt, Germany) mass spectrometer (MS) consisting of an ESI source and a triple quadrupole-ion trap mass analyzer, which was controlled by the Analyst 5.1 software. An Aqua C18 125 Å column (5 μm, 250 × 4.6 mm I.D.; Phenomenex) thermostated at 35 °C was used. The solvents were: (A) 0.1 % formic acid, and (B) acetonitrile.

The elution gradient established was isocratic 15 % B for 5 min, 15–20 % B over 5 min, 20–35 % B over 10 min, 35–50 % B over 10 min, 50–60 % B over 2 min, isocratic 60 % B for 5 min and re-equilibration the column, using a flow rate of 0.5 mL/min. Double online detection was carried out in the DAD using 280 nm and 370 nm as preferred wavelengths and in the MS operated in the negative ion mode. Spectra were recorded between *m/z* 100 and 1000. Zero grade air served as the nebulizer gas (30 psi) and as turbo gas (400 °C) for solvent drying (40 psi). Nitrogen served as the curtain (20 psi) and collision gas (medium). Both quadrupols were set at unit resolution and EMS and EPI analyses were also performed. The EMS parameters were: ion spray voltage 4500 V, DP −50 V, EP −6 V, CE −10 V and cell exit potential (CXP) -3 V, whereas EPI settings were: DP −50 V, EP −6 V, CE −25 V and CES 0 V.

The phenolic compounds present in the samples were identified according to their UV and mass spectra and by comparison with commercial standards when available.

### Statistical analysis

Data are expressed as mean ± SD from three independent experiments. The analysis average of the treatment using multiple comparisons was determined by using Duncan’s multiple range tests, and the data were compared using the *p* values: *p* < 0.05 was considered statistically significant. The least significant difference (LSD) was used to determine the difference between the methods used to the investigation of the various antioxidant capacities. The statistical analysis of differences between various treatments on cells was determined by the Student’s *t*-test. Values of *p* < 0.05 were considered statistically significant. Statistical analysis was performed with GraphPad Prism 6 software (GraphPad Software, San Diego, California, USA).

## Results

### Total antioxidant activity

The IC_50_ values of leaves, flowers and tubers extracts and positive control (trolox) were calculated in the present study and are depicted in Table 1. The extracts scavenged the ABTS and DPPH radicals in a concentration dependent manner. For DPPH assay, ethanolic extracts from flowers and leaves (FEE and LEE respectively) showed the best activity (IC_50_ = 28.4 ± 0.85 μg/mL for FEE and IC_50_ = 55.9 ± 1.55 μg/mL for LEE) compared to the other extracts (*p* < 0.05). Likewise, for ABTS radical scavenging assay, FEE and LEE showed an IC_50_ of 33.1 ± 1.55 μg/mL and 74.5 ± 7.77 μg/mL, respectively. The water extracts of tubers showed the smallest scavenging capacity.

**Table 1:** Free radical-scavenging content of *A. microcarpus* extracts

Extracts		IC_50_ values (μg/mL)	
		ABTS scavenging	DPPH scavenging
Leaves	Aqueous	174.35 ± 7.99^e^	134.75 ± 7.85^b^
	Ethanolic	74.5 ± 7.77^b^	55.9 ± 1.55^a^
	Methanolic	131.1 ± 1.27^d^	140.85 ± 0.49^b^
Flowers	Aqueous	126.4 ± 5.09^c^	108 ± 2.83^b^
	Ethanolic	33.1 ± 1.55^a^	28.4 ± 0.85^a^
	Methanolic	107.6 ± 1.27^de^	113.25 ± 1.77^b^
Tubers	Aqueous	720.45 ± 13.5^h^	670.4 ± 27.72^e^
	Ethanolic	257.75 ± 10.96^f^	360 ± 56.57^c^
	Methanolic	680.25 ± 14.35^g^	579 ± 29.7^d^
Trolox		3.4 ± 0.3	3.2 ± 0.4

*Note*: The results are expressed as IC_50_ values (μg/mL). The data are given as mean ± standard deviation (SD) of triplicate experiments. The statistical comparison between values from the different plant extracts applied using the post hoc Duncan test. Means followed by distinct letters in the same column were found to be significantly different (*p* < 0.05)

The IC_50_ values are high if compared with the standard (IC_50_ ~ 3.3 μg/mL). The crude extracts examined probably contain pro-oxidants agents which may compete with the antioxidants in the reaction with ABTS and DPPH radicals.

### Phenolic and flavonoid contents

The phenolic and flavonoid amount of each extract is showed in Table 2. For each part of the plant, the best results were obtained with ethanolic extracts with a total phenolic contents of 39.35 ± 4.2, 54.44 ± 13.6 and 68.62 ± 9.8 mg GAE/g dw, for TEE, LEE and FEE respectively. Tubers extracts displayed the lowest level of total phenolics.

**Table 2:** Polyphenol and flavonoid content in leaves, flowers and roots extracts from *A. microcarpus*

Extracts		Total polyphenols^(*)^	Flavonoids^(**)^
Leaves	Aqueous	36.83 ± 0.6^d^	5.90 ± 2.52^c^
	Ethanolic	54.44 ± 13.6^e^	31.13 ± 1.96^g^
	Methanolic	35.48 ± 0.4^d^	17.27 ± 1.28^e^
Flowers	Aqueous	36.58 ± 1.2^d^	3.33 ± 0.41^b^
	Ethanolic	68.62 ± 9.8^f^	27.28 ± 2.33^f^
	Methanolic	26.37 ± 1.2^c^	11.43 ± 1.07^d^
Tubers	Aqueous	5.10 ± 0.5^a^	1.99 ± 0.42^ab^
	Ethanolic	39.35 ± 4.2^d^	1.4 ± 0.33^a^
	Methanolic	15.31 ± 7.8^b^	3.94 ± 1.05^b^

Data are expressed as mean of three measurements ± standard deviation

(*) mg GAE/g of dry weight

(**) mg QE/g of dry weight

*Note*: The data are given as mean ± standard deviation (SD) of triplicate experiments. The statistical comparison between values from the different plant extracts applied using the post hoc Duncan test. Means followed by distinct letters in the same column were found to be significantly different (*p* < 0.05)

There were significant differences in the plant extracts in terms of contents of total flavonoid, which varied from 1.4 (TEE) to 31.13 (LEE) mg QE/g dw. The highest flavonoid content was found in LEE (31.13 ± 1.96 mg QE/g dw) followed by FEE (27.28 ± 2.33 mg QE/g dw). The results showed that tubers are the part of the plant with lowest polyphenol and flavonoids contents.

### Tyrosinase inhibition

Table 3 shows the inhibition of tyrosinase activity by extracts and kojic acid (positive control) at 0.2 mg/mL. The tyrosinase inhibition ranges of AE, EE and ME were 6.55–10.65, 8.4–40.25, and 2.25–20.4 %, respectively.

**Table 3:** Inhibition of tyrosinase by *A. microcarpus* extracts

Extracts		% Inhibition at 0.2 mg/mL
Leaves	Aqueous	9.85 ± 0.21^b^
	Ethanolic	29.9 ± 0.14^d^
	Methanolic	20.4 ± 1.4^c^
Flowers	Aqueous	6.55 ± 0.21^b^
	Ethanolic	40.25 ± 4.4^e^
	Methanolic	13.9 ± 2.4^b^
Tubers	Aqueous	10.65 ± 1.34^b^
	Ethanolic	8.4 ± 1.3^b^
	Methanolic	2.25 ± 1^a^
Kojic Acid		97.4 ± 2.8

*Note*: The data are given as mean ± standard deviation (SD) of triplicate experiments. The statistical comparison between values from the different plant extracts applied using the post hoc Duncan test. Means followed by distinct letters in the same column were found to be significantly different (*p* < 0.05)

**Fig. 1:**
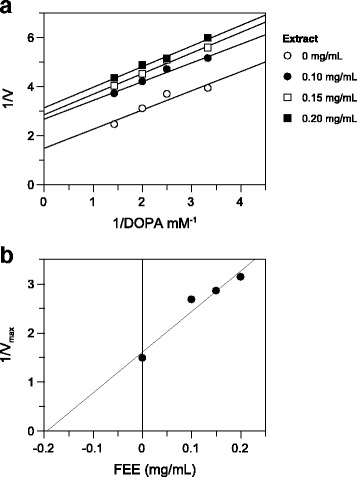
Inhibition of tyrosinase activity by *A. microcarpus* flowers extract. **a** Lineweaver-Burk plot for inhibition of ethanol extract on mushroom tyrosinase activity using l-DOPA as substrate. Reaction mixtures contained mushroom tyrosinase in 25 mM phosphate buffer (pH 6.8) and l-DOPA, in the absence or presence of extract at different extract concentrations. **b** Replot of the 1 ⁄V_max_ values versus extract concentration

The results indicated that the ethanolic extracts showed good activity, while the water and methanolic extracts showed only moderate activity at the concentration tested. The attention was, therefore, focused on ethanolic extract from flowers that show the best enzyme inhibition when compared with other extracts (*p* < 0.05). An IC_50_ value of 0.27 mg/mL was determined and the kinetic behaviour of tyrosinase at different concentration of l-DOPA and FEE was also investigated (Fig. 1). The Lineweaver-Burk plot gave a family of parallel straight lines with the same slope (Fig. 1a). With the increasing of the inhibitor concentration, the values of both K_m_ and V_max_ are reduced, while the ratio K_m_/V_max_ remains quite the same. The slopes are independent from the concentration of FEE, which indicates that the compound is an uncompetitive inhibitor of the enzyme. The inhibition constant (Ki) of 0.19 mg/mL was obtained from a plot of the vertical intercept (1/V_max_) versus the inhibitor concentration (Fig. 1b).

### Cell viability

**Fig. 2:**
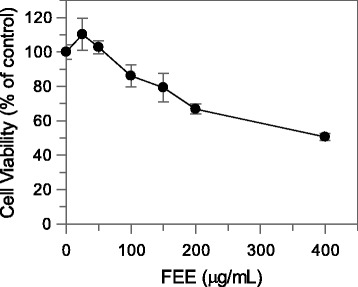
Effect of *A. microcarpus* flowers extract on cell viability in B16F10 melanoma cells. After 48 h incubation with FEE, cell viability was determined by MTT assay. Data are expressed as mean ± SD from three independent experiments

The FEE showed the best scavenging capacity and mushroom tyrosinase inhibitory activity, so its ability to affect the viability of B16F10 melanoma cells was also evaluated. B16-F10 cells were treated with EE of flowers at concentrations ranging from 5 to 400 μg/mL for 48 h at 37 °C and were examined using the MTT test. The results showed that FEE did not have a significant cytotoxic effect until 150 μg/mL (viability of 80 %), while 200 and 400 μg/mL resulted in a loss of viability of 35 and 50 % respectively (Fig. 2). Thus, further experiments using up to 150 μg/mL extract concentration were performed.

### Effect of FEE on intracellular tyrosinase activity and melanin content in melanoma cells

**Fig. 3:**
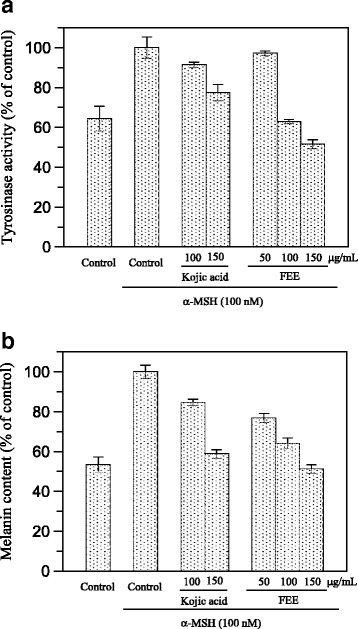
Effect of flowers extract on B16F10 melanoma cells. Tyrosinase activity (**a**) and melanin production (**b**) are expressed as percentage of the control and the effects of FEE were compared with Kojic acid as standard inhibitor

To obtain information about the inhibiting potency of FEE in the cellular model, the inhibitory effect of the extract on the tyrosinase activity of B16F10 cells treated with 100 nM *α*-MSH was examined. Upon exposure to *α*-MSH alone, the tyrosinase activity was significantly increased, compared to untreated cells (Fig. 3a). After 48 h of incubation with FEE, tyrosinase inhibition at 50, 100 and 150 μg/mL was 2.85 ± 1.2, 37.14 ± 1.1 and 48.4 ± 2.26 % respectively. Thus, FEE significantly reduced the tyrosinase activity in murine cells in a concentration-dependent manner. The inhibitory effect of the ethanolic extract was even much stronger than that of kojic acid, the positive control, that showed a tyrosinase inhibition of 8.65 ± 1.44 and 22.61 ± 4.13 % at 100 and 150 μg/mL respectively.

The melanin content of *α*-MSH-stimulated B16F10 cells in presence of the extract was also evaluated. Fig. 3b shows that FEE also reduced cellular melanin in a concentration-dependent manner. Comparing the results obtained with the extracts and with kojic acid at the same concentrations of 100 μg/mL and 150 μg/mL, FEE exerted the highest cellular antimelanogenesis effect with an inhibition of 35.92 ± 2.77 and 48.77 ± 2.11 % versus 15.45 ± 0.64 and 41.2 ± 2.12 % of the standard inhibitor.

### DOPA staining

**Fig. 4:**
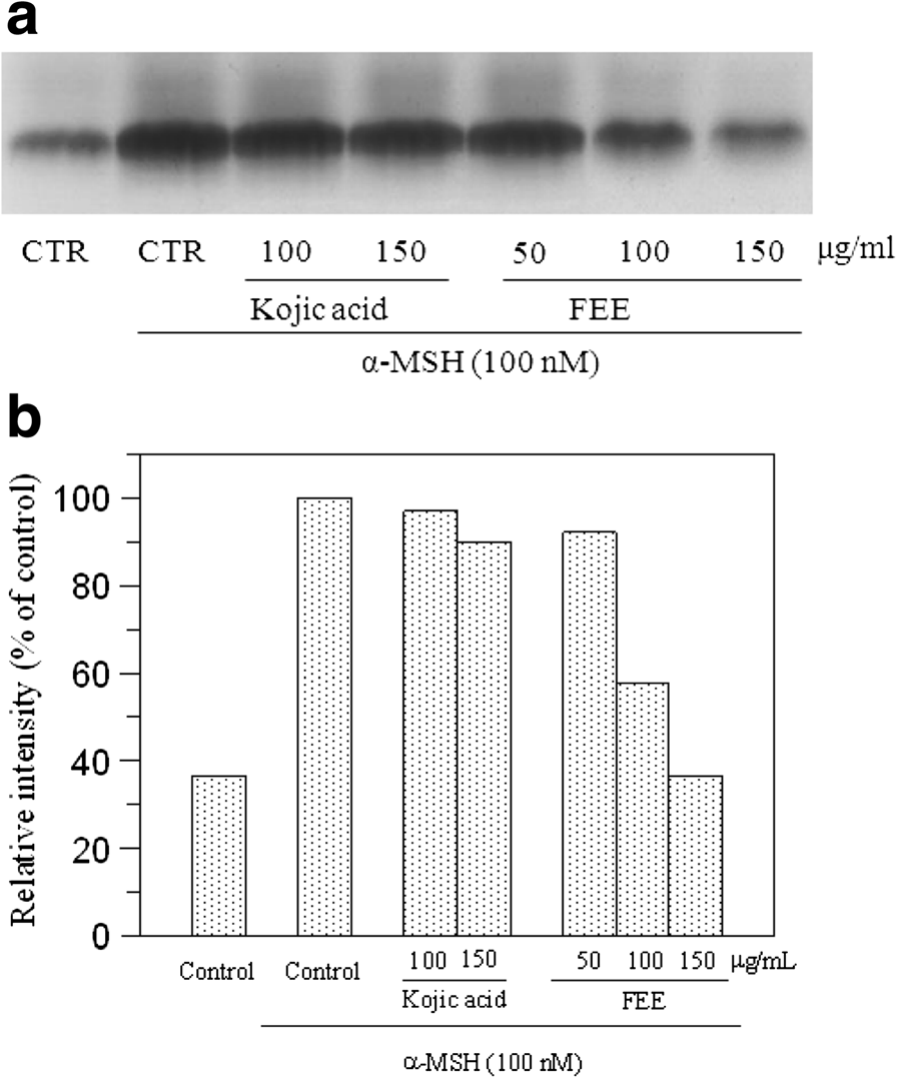
Effect of flowers extract on B16F10 cells by L-DOPA staining. Tyrosinase activity was estimated by zymography (**a**) and the relative intensity of bands was determined with ImageJ software (**b**)

Effect of FFE on the intracellular tyrosinase activity was also confirmed by tyrosinase zymography. B16F10 cells were treated with *α*-MSH alone or *α*-MSH plus substances (extract or kojic acid). Tyrosinase activity in non-treated cells was very low while *α*-MSH-stimulated cells showed dark band with higher activity. FEE seemed to be almost ineffective at the concentration of 50 μg/mL while at 100 and 150 μg/mL of extracts, activity of tyrosinase decreased and lighter bands were observed (Fig. 4a and b). Results are in agreement with the data of intracellular tyrosinase inhibition, confirming the anti-melanogenic effect of the extract, ever better than kojic acid.

### Characterization of phenolic compounds in A. microcarpus flowers extract

**Fig. 5:**
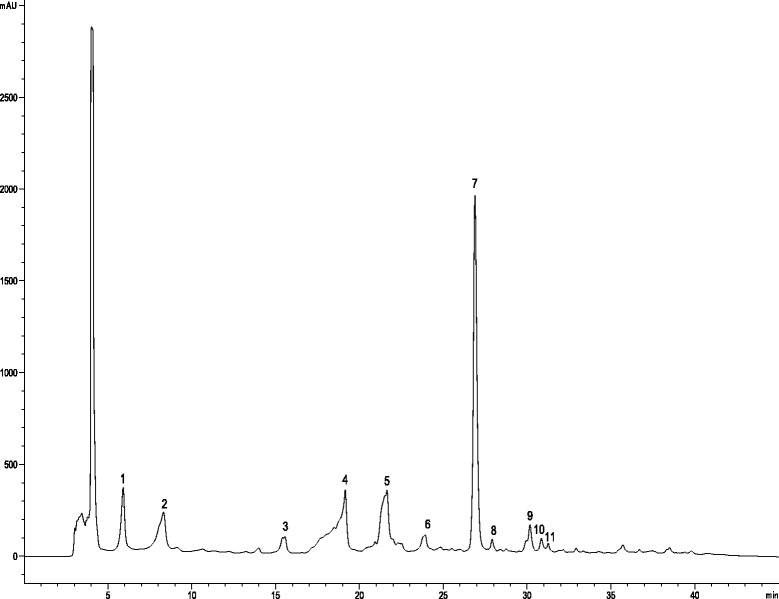
HPLC chromatogram of FEE recorded at 330 nm for phenolic compounds. Peak identification is given in Table 4

The HPLC phenolic profile of *A. microcarpus* FEE is shown in Fig. 5 and identification for each peak of the phenolic compounds detected are listed in Table 4.

**Table 4:** Identification of polyphenolic compounds in *A. microcarpus* flowers ethanolic extract by HPLC-DAD-ESI/MS analysis

Peak	Rt (min)	λmax (nm)	Pseudomolecular ion [M-H]^−^ (*m/z*)	MS^2^ (*m/z*), (%)	Tentative identification
1	5.9	326	353	191 (100)	3-*O*-caffeoylquinic acid
				353 (94)	
				179 (71)	
				135 (48)	
				173 (9)	
2	8.3	326	353	191 (100)	5-*O*-caffeoylquinic acid
3	15.4	346	447	447 (100)	Luteolin-6-*C*-glucoside
				357 (98)	
				327 (97)	
				285 (25)	
				429 (20)	
4	19	340	447	447 (100)	Luteolin-*O*-hexoside
				285 (40)	
5	21.5	338	447	285 (100)	Luteolin-7-*O*-glucoside
6	23.9	338	489	285 (100)	Luteolin *O*-acetylglucoside
7	26.8	345	285	285 (100)	Luteolin
8	27.9	324	593	285 (100)	Luteolin *O*-deoxyhexosylhexoside
9	29.9	288	271	151 (100)	Naringenin
				119 (56)	
10	30.2	338	269	269 (100)	Apigenin
				151 (36)	
11	30.8	348	299	300 (100)	Methyl-luteolin
				285 (88)	

Peaks 1 and 2 were tentatively assigned as 3-*O*-caffeoylquinic acid and 5-*O*-caffeoylquinic acid based on their chromatographic characteristics and fragmentation patterns according to the identification keys described by Clifford [25]. The identity of 5-*O*-caffeoylquinic acid was further confirmed by comparison with an authentic standard. Peak 3 showed a molecular ion [M-H]^−^ at *m/z* 447 giving place to four MS^2^ fragment ions, a major one at *m/z* 357 [M-H-90]^−^, and other three at *m/z* 327 [M-H-120]^−^, m/z 285 [M-H-162]^−^ and at *m/z* 429 [M-H-18]^−^.

This fragmentation pattern is characteristic of *C*-glycosylated flavones at C-6/C-8, according to the data previously reported [26]. Thus, the peak was identified as luteolin-6-*C*-glucoside, which was also confirmed by comparison with a standard. The same pseudomolecular ion [M-H]^−^ was found for peaks 4 and 5 ([M-H]^−^ at *m/z* 447), but in both cases only a characteristic fragment at *m/z* 285 [M-162]^−^ was produced, indicating the correspondence to *O*-hexosides. They were tentatively assigned as luteolin *O*-hexoside and luteolin 7-*O*-glucoside, respectively; the identity of this latter was confirmed by comparison with a standard. Peaks 6 and 8 presented pseudomolecular [M-H]^−^ ions at *m/z* 489 and 593, respectively, and were identified as luteolin derivatives owing to the production of a common luteolin MS^2^ fragment at *m/z* 285. They were assigned as luteolin *O*-acetylglucoside and luteolin *O*-deoxyhesylhexoside, respectively, based on the losses of 204 mu (162 + 42 mu) and 380 mu (146 + 162 mu) to produce the MS^2^ fragment. The remaining four peaks were assigned as different flavonoid aglycone based on their UV spectra and mass characteristics. Thus, the majority peak 7 was identified as luteolin, peak 9 as the flavanone narigenin, and peak 10 as apigenin, as also confirmed by comparison with standards. Finally, peak 11, with a pseudomolecular ion [M − H]^−^ at *m/z* 299 releasing a fragment at *m/z* 285 from the loss of a methyl residue (14 mu), was assigned as methyl-luteolin.

## Discussion

The interest in finding novel antimelanogenic agents from natural sources with antioxidant activity is of great interest. Since the key role of tyrosinase in melanin pathway, research of molecules that inhibit tyrosinase have become increasingly important for medicinal and cosmetic products that may be used as powerful skin-whitening agents for treating skin disorders.

In this study the antioxidant capacity of different extracts of *A. microcarpus* was analyzed and their effects on the tyrosinase activity and melanin synthesis was evaluated.

*A. microcarpus* extracts showed significant antioxidant activity. In particular, the ethanolic extract of the flowers showed the highest scavenging activity, which could be attributed mainly to its high levels of total polyphenols and flavonoids. Inhibitory effects of *A. microcarpus* extracts on mushroom tyrosinase were evaluated, and also in this case FEE revealed the highest inhibitory activity with an uncompetitive mode of inhibition. Antimelanogenic effect of this extract was confirmed in assays in B16F10 cells, being even more active than the positive control (kojic acid).

It is well known that polyphenols, and namely flavonoids, behave as inhibitors of ROS generation and could be responsible for the antimelanogenic activity of plant extracts [27–30].

The major compound in the extract was the aglycone luteolin, a compound that in a previous study was reported to show whitening activity. This compound did not inhibit directly tyrosinase and its activity was attributed to the inhibition of adenyl cyclase involved in the signalling pathway of α-MSH in B16F10 melanoma cells. In α-MSH-stimulated B16 melanoma cells, luteolin inhibited both tyrosinase activity and melanin production in a concentration-dependent manner [27].

This might explain the greater inhibition of FEE on B16F10 cells compared to the effect towards mushroom tyrosinase.

The inhibition of tyrosinase has an important role in order to prevent melanin accumulation in skin. Therefore, tyrosinase inhibitors are an attractive target in cosmetics and treatments for pigmentation disorders. The *A. microcarpus* extract may be used for the production of herbal preparations containing phytochemicals with significant bioactivity or as a source of inspiration for the development of new drug with less toxic side effects.

## Conclusion

In conclusion, our results demonstrated that ethanolic flower extract of *A. microcarpus* has strong tyrosinase inhibitory activity. This effect is even better than the standard inhibitor in cellular system. Moreover, it also shows the highest scavenging activity, which could be attributed mainly to its high levels of total polyphenols and flavonoids. These results suggest that FEE may be helpful such as source of bioactive compounds for controlling hyperpigmentation and as skin whitening agents.

## Abbreviations

ABTS: 2,2′-azinobis-(3-ethylbenzothiazoline-6-sulfonic acid)
BSA: Bovine serum albumin
DOPA: 3,4-dihydroxyphenylalanine
DPPH: 2,2-diphenyl-1-picrylhydrazyl radical
GAE: Gallic acid equivalent
MTT: 3-(4,5-dimethylthiazol-2-yl)-2,5-diphenyltetrazolium bromide
QE: Quercetin equivalent
Trolox: 6-hydroxy-2,5,7,8-tetramethylchromane-2-carboxylic acid
*α*-MSH: α-melanocyte stimulating hormone
